# Development of student self-efficacy in innovative fieldwork placements: A qualitative study

**DOI:** 10.1177/03080226251403332

**Published:** 2025-12-26

**Authors:** Mackenzie Cheng, Felicity Niles-Williams, Andrea Duncan, Anne W. Hunt

**Affiliations:** 1 Department of Occupational Science & Occupational Therapy, University of Toronto, Toronto, ON, Canada; 2 Department of Occupational Science & Occupational Therapy, Rehabilitation Sciences Institute, University of Toronto, Toronto, ON, Canada

**Keywords:** Occupational therapy education, experiential learning, competency development, LEAP placements, self-efficacy

## Abstract

**Introduction:**

Self-efficacy plays an integral role in the development of occupational therapy students’ clinical competency during fieldwork education. However, there is still a lack of knowledge surrounding this topic within the context of non-traditional fieldwork education. This study aims to investigate and make meaning of the experiences of occupational therapy students’ and their perceived development of self-efficacy in non-traditional fieldwork placements, known as LEAP placements.

**Method:**

This qualitative phenomenology study, through a descriptive lens, explored sixteen occupational therapy students’ experiences using thematic analysis.

**Findings:**

Self-efficacy during LEAP placements was shaped by several factors: a lack of role clarity detracted from development, while the opportunity for self-direction enhanced it. The level of support, preceptor role and availability, perceived value of the learning experience, and placement environment both enhanced and detracted from self-efficacy development.

**Conclusion:**

This study provides an increased understanding of self-efficacy development among occupational therapy students and innovative practices within LEAP placements. Students’ perceptions of self-efficacy development is shaped by complex and nuanced factors.

## Introduction

Many occupational therapy (OT) programs are experiencing increases in the use of innovative and non-traditional fieldwork (FW) placements (Gill et al., 2024). As a result, there is a need to understand more about how these placements contribute to students’ learning and development of self-efficacy. This study aims to investigate and make meaning of OT students’ experiences of self-efficacy development while completing a LEAP fieldwork placement (Leadership, Role Emerging, Advocacy & Program Development).

## Literature review

The acquisition of clinical skills is crucial for student occupational therapists as they prepare to enter practice (Bradley et al., 2013; Thomas and Penman, 2019). Fieldwork placements enable OT students to gain experience and solidify concepts within a clinical setting; therefore they are a fundamental part of professional education (Bonello, 2001; Cohn and Crist, 1995). Fieldwork placements are structured to allow students to build their self-efficacy, clinical skills and competencies. Various studies on self-efficacy, as impacted by student fieldwork placements, have noted its positive effects on clinical performance (Andonian, 2013; Derdall et al., 2002; Hussain et al., 2018). Additionally, there has been a myriad of research exploring fieldwork experiences and professional development among student OTs, preceptors, and faculty educators (Bonello, 2001; Dancza et al., 2019; Karp, 2020). However, there is limited research on the development of student-perceived self-efficacy within the context of non-traditional and innovative fieldwork placements (Hussain et al., 2018). These placements have been termed LEAP placements, which stands for Leadership, Role-Emerging, Advocacy, and Program Development (Barker and Duncan, 2020).

LEAP placements often occur in non-traditional settings, including those in which there is no onsite occupational therapist. Students are tasked with non-clinical learning opportunities that may involve activities such as the development of the occupational therapy role within a non-traditional organization, completing program evaluations, population needs analyses, environmental scans, developing advocacy programs, or client educational materials. LEAP placements are typically full-time learning experiences for periods of 6–8 weeks. Students may be supervised onsite by a non-occupational therapist and meet regularly with an off-site occupational therapy preceptor who provides profession-specific supervision (Barker and Duncan, 2020). Non-traditional placements, like LEAP placements, have existed for well over two decades within Occupational Therapy education, but became more common during the COVID-19 pandemic (Gill et al., 2024) due to the limitations on clinical sites at that time.

Gill et al. (2024) described the proliferation of LEAP placements that emerged due to pandemic-related practice shifts. These included placements that were virtual (e.g. telehealth), administrative (e.g. policy development) or role emerging (e.g. art or nature-based approaches). Shifts toward more innovative occupational therapy practice and student placements call for further research to gain a comprehensive understanding of OT students’ development of self-efficacy in these less familiar settings. Self-efficacy has been described as “a belief in one’s capabilities to tackle tasks successfully” (Bandura, 2001) and the belief in one’s abilities and the effort to use them, which can contribute to achieving desired outcomes (Burić and Moe, 2020). Self-efficacy can influence cognitive, motivational, emotional, and decision-making processes, affecting performance and interpretations of success and failure (Bandura, 2012).

The literature identified four factors in developing self-efficacy. First is mastery of experiences, where resilience and learning from successes and failures guide future endeavors (Bandura, 2012). Second is vicarious experiences/social modeling, learning from observing others (Bandura, 1977). Third is verbal persuasion, where external validation and feedback encourage goal pursuit and personal improvement. Finally, psychological states reflect mental and emotional well-being, and conversely, stress can lead to fear, anxiety, and depression, both of which can influence self-efficacy (Bandura, 2012). Efficacy beliefs improve with self-regulation and viewing challenges as learning opportunities, reframing outlooks on personal development. For instance, fear and self-doubt can lead to avoidance behaviors, whereas confidence can motivate and initiate engagement (Bandura, 1977).

The current health literature contains much informal dialogue about self-efficacy as an integral factor in successful student fieldwork placements (Derdall et al., 2002; Hussain et al., 2018). Studies have shown that physiotherapy students with higher self-efficacy perform better in clinical placements than those with lower self-efficacy (Andonian, 2013; Utsey, 2006). Additionally, a study by Swinehart and Meyers (1993) has shown that occupational therapists and student occupational therapists have identified self-efficacy as one of the main purposes of fieldwork placements. However, self-efficacy; and its relation to OT student performance within the context of innovative fieldwork placements such as LEAP, have yet to be fully researched. Exploring the perspectives of occupational therapy students during LEAP placements may provide some understanding about how these innovative experiences affected self-efficacy development.

In our study, we sought to answer the question: *How did occupational therapy students experience the development of self-efficacy through participation in a LEAP fieldwork placement?*

## Method

A phenomenological study, adopting a descriptive approach, was used. Phenomenology was used to uncover the intricacies of unique phenomena, focusing on how individuals describe these experiences. This descriptive approach, rooted within relativist ontology and subjectivist epistemology, considers multiple perspectives equally without the need to seek out one truth or reality and acknowledges the co-construction of knowledge between participants and researchers (Ponterotto, 2005). It utilizes our role as researchers to comprehensively interpret these descriptions while bracketing our pre-assumptions to view the phenomenon through an unbiased lens (Reiners, 2012). This study was approved by the University of Toronto’s research ethics board (REB #40783; original approval date March 3, 2021).

### Positionality

As Canadian clinical occupational therapists and university faculty engaged in the study of fieldwork education, we recognize the influence of our personal, professional and institutional perspectives on this research. We acknowledge that our experiences, values and perspectives shape how we view fieldwork education. Our university has been a champion over the past decade for LEAP placements and they are an integral part of our program today. We value these learning experiences as an integral part of occupational therapy education and yet recognize the need to test our values and views by conducting foundational research to support or refute these beliefs. By maintaining transparency in our positionality, we aim to enhance the credibility and trustworthiness of our research through improved understanding of approaches in occupational therapy fieldwork education.

### Design

This study is a secondary analysis of data collected from a larger research study (Gill et al., 2024). The original study used Yin’s (2012) case study method to identify and describe fieldwork innovations that occurred during the early years of the COVID-19 pandemic. Yin’s case study method enables the study of real-life contexts that have historical significance by conducting cross-case analyses to identify common and differing factors across multiple cases represented in the data (Gill et al., 2024; Yin, 2012). Recently graduated occupational therapists participated in in-depth interviews about their experiences in innovative fieldwork placements. Among other things, interviews included questions about their perspectives on the development of self-efficacy when they were students (e.g. learning from success/failures in less familiar environments, validation from preceptors, impact of these factors on performance). Utilizing secondary data generates new understandings from already produced data (Irwin, 2013) and helps expand on topics more thoroughly than the original study.

### Study sample/participants

Participants were recruited via email. Inclusion criteria stipulated that participants must have successfully completed the OT program at the time of the initiation of the primary study, had a minimum of 3 months’ work experience as an OT, and identified that they completed a LEAP placement between March 2020 and December 2020. Exclusion criteria were failure to provide informed consent and self-reported inability to provide informative feedback regarding their experiences of a LEAP placement. All participants provided informed written consent.

### Data collection

The data consisted of transcripts of 16 semi-structured interviews with study participants. Interviews were conducted via Zoom (2022). Participants’ reflections were captured in 30–60 minute interviews and the audio data were transcribed verbatim.

### Data analysis

Two members of the research team, who were not involved in the original study, began by reviewing all the data. The goal of this initial review was to become familiar with the data and identify the phenomenon experienced by the participants (Monaro et al., 2022). Through reflective journaling and research team discussion, it was agreed that among the multitude of potential constructs for further exploration, self-efficacy was the most prominent phenomenon reported by the participants. More specifically, anecdotes and broad ideas were identified that reflected the four self-efficacy factors (e.g. mastery of experiences, vicarious experiences, verbal persuasion, and psychological state) and discussed by the research team to ensure these data were representative of self-efficacy. Anecdotes were then organized into potential themes relating to participants’ experiences and their self-efficacy development. Deductive thematic analysis was used to analyze transcribed data from the original study based on the four self-efficacy factors. During data analysis, two researchers met weekly to check among each other to ensure consistency between coding categories and themes and to account for any pre-assumptions through bracketing. Inconsistencies or disagreements were brought to two other members of the research team for further analysis. The whole research team met biweekly until agreement was reached on themes and their descriptions. The qualitative coding software (NVivo 12) was used to support data analysis.

## Findings

Participants were 16 occupational therapists who identified as completing a LEAP placement from March 2020 to December 2020 as part of their MScOT program. All participants were recent graduates (i.e. within 2 years post-graduate). Four main themes were constructed from the data analysis that describe students’ perceptions of self-efficacy during LEAP placements: (1) Being on my own, (2) Preceptor perception and support, (3) Perceived learning, and (4) Fieldwork environment. Each main theme consisted of several sub-themes (Table 1).

**Table 1.** Findings: Identified themes and subthemes.

Themes	Being on my own	Preceptor perception and support	Perceived learning	Fieldwork environment
Subthemes	Student clarity of their own role	Preceptor role	Passions and interests	Hybrid environment
	Staff understanding of student role	Preceptor availability	Soft skills development	Innovation
	Student self-direction	Other resources offered	Limited learning opportunities for hard skills development	

### Being on my own

“Being on my own” is how participants described their general sense of their position as the student OT in a LEAP fieldwork placement. Participants reflected that their self-efficacy was impacted by their perceptions of their responsibilities and heightened expectations of themselves in a LEAP placement. Factors affecting this theme include the student’s clarity of their own role, other staff’s understanding of the student’s role as an OT, and the student’s ability to work in a self-directed manner.

#### Student’s clarity of their own role

Some participants who lacked clarity regarding their role or who were faced with other colleagues who were unsure of their role experienced negative impacts to their self-efficacy due to the inability to feel comfortable initially while on placement.

*I feel like there was a lot of work that needed to be done to make it feel comfortable because it– there was really no clear spot for me to fit in.*—*1001*

I didn’t have too much mentorship. . .I was like, “Is this– is what I’m doing really OT? Is this part of my role?” And I didn’t really have anyone to approach.—1005

#### Student self-direction

Self-direction appeared to be a factor that positively influenced participants’ self-efficacy. Many participants identified self-directed learning as an essential skill, and reflected on how it positively impacted their ability to learn and feel confident during placement. Depending on the individual, some were able to appreciate a more self-directed learning environment and found positive development in their self-efficacy, while others found it more difficult being on their own and experienced a negative impact on their self-efficacy.

*“[. . .] you have to take the initiative to reach out to your preceptor or to other professionals in the organization if you wanna learn more about what did they do. And shadowing and those kinds of things, so, I think one, one of those skills is that self-directed learning and taking initiative to develop your own skill set which will be something you’ll be continuously doing as an OT*”—1013

*I was kind of thrown in. “It’s okay,” I was telling myself, “You’re uncomfortable with ambiguity, but it’s okay. You can figure it out.”*—*1007*

### Preceptor perception and support

Perception of how much support they had while on their fieldwork placements was another influential piece related to participant self-efficacy. Factors affecting this theme involved the preceptor role, whether the preceptor was an occupational therapist by background or worked in another profession, preceptor availability; how many meetings occurred during fieldwork placement and whether the participant felt that they could reach out to their preceptor for support, and other resources offered at fieldwork, such as the presence of a partner, other staff or supervisors.

#### Preceptor role

Some participants with preceptors who were not OTs found it more challenging to understand their role as students, experiencing uncertainty regarding OT treatment decisions. Other participants provided insights into the challenges of having an off-site OT preceptor who was not closely involved with the LEAP placement organization, limited their ability to learn from them, leaving the participant to be more in charge of their learning experience.

*. . .my off-site preceptor works in the community, she doesn’t work with the non-profit, and she didn’t always know how to address certain problems when the client doesn’t have the money to buy some random thing which would be super helpful for them, or she’s not a case manager so she doesn’t know how to support from an OT perspective in that way.*—*1001*

#### Preceptor availability

Participants reported self-efficacy varied depending on their perception of preceptor availability. Participants who needed assistance but were not able to meet frequently with their preceptor experienced challenges regarding their feelings of competency on fieldwork placement. On the other hand, participants who perceived their support from their preceptor to be sufficient did not experience similar challenges.

*We met one hour or two hours once a week on Fridays or something. Something like that. It was once a week. And sometimes it felt like it wasn’t enough.*—*1002*

*I think for me and my partner, because we were both very independent and very organized I would say we found that the once a week meetings were all that we needed because me and [my placement partner] we would be meeting and talking, like, every day almost. But in terms of our weekly with the preceptors, we felt – it was just right because when we weren’t meeting with her, we were working by ourselves, meeting deadlines, and doing everything by ourselves. Right? But I think that comes with us having been, so organized and the fact that we got along really well and we had the same goals and attacked the project with, the same mindset. So I, personally, found that that level of supervision was fine for us*.—*1012*

There are also instances of participants wishing to have more independence during their placement as they find they were not able to develop the self-efficacy needed in their own skills as an OT.

*I was kind of also always under supervision, but sometimes, my, my OT was great, my preceptor, but, um, I also didn’t develop that confidence of, of like, “Okay, you’re your own practitioner. You’re making the calls,”*—*1013*

#### Other resources offered

Other than preceptor support, participants’ self-efficacy appeared to be affected by the presence of a fieldwork partner and other staff who were able to step in and guide them when needed. Participants spoke about their experience being supported by others in their fieldwork placement despite their preceptor’s absence, in addition to the advantages of having multiple viewpoints from various individuals.

*I feel like if it was completely alone, I would have felt very lost almost. And even though I did have the guidance of my preceptors, I think it was really good to be reassured by your partner as well, about things. And I think that was something that I found challenging, which I was able to find a support for.*—*1004*

### Perceived learning

This theme reflects participants’ subjective interpretations of the knowledge, skills, and competency development they acquired while on a LEAP placement. Factors affecting this theme include unique passions and interests, the hard and soft skills development and any opportunities, barriers, or challenges to learning.

#### Passions and interests

Participants desired and sought learning opportunities that aligned with the setting they wanted to work in once they graduated. Participants were able to recognize that their self-efficacy was greatly impacted when their LEAP placement was in their desired setting and when they learned skills related to their goals and passions. Participant self-efficacy appeared to be negatively impacted when they were in a setting where they felt they did not have foundational skills to perform. It became apparent that when participants could not connect with or had a lack of interest in the practice setting, they had difficulty motivating themselves to explore the learning opportunities the placement had to offer.

*I wanted to work in a hospital. But I was fortunate to have face-to-face in-person experience. And I think that’s tremendously valuable. I think, had I not been redeployed, I would have felt maybe a little bit at a disadvantage coming out of my program. But that’s because my goal is, you know, to work in a hospital. But for someone who has a goal in research or a goal in more of a mental health capacity or developing a program, program management, a managerial position I don’t think they would have felt the same way. –1016*

Participant self-efficacy may have been negatively impacted because they believed they were not developing the skills that would support them in client facing positions.

*I found that when I actually had real, in-person clinical experience [for my fourth placement]. . . I found that I cared a lot more. . ., I found it a lot more relevant to what I was actually doing versus, my [third placement-LEAP virtual] placement was just like I was doing it to do it. I was essentially doing it to graduate or get the credit. Right? But I felt that for my fourth placement, the resources and the things I was working on actually contributed to my clinical experience essentially.*—*1012*

#### Soft skills development

A consistent point of discussion among participants was how they compared the skills they learned during a LEAP placement with those gained in traditional placement settings. Participants made note that they developed “soft skills” during their LEAP placements and emphasized how, during innovative placements, they “developed a lot of communication skills, both verbal and nonverbal” (1009), “time management or work management” (1009) “organizational skills” (1011), and “reflection skills” (1011). Additionally, participants indicated that “collaborating with [their] peers and brainstorming and problem-solving” (1011) were other skills developed. Participants highlighted characteristics like “critical thinking” (1014), “creativity” (1014), “flexibility” (1016), and having to be “adaptable” (1011) were necessary to build and support their learning. Participants saw the value in soft skills learned during a LEAP placement and may have positively impacted their self-efficacy because of its contribution to their professional values.

*“for my international as well as my virtual placement, I definitely learned more soft skills. I think those skills helped shape more what I value as a practitioner, what’s important”*—*1003*

#### Limited learning opportunities for hard skills development

Participants reported a stronger sense of self-efficacy when they learned and developed “hard skills,” during traditional fieldwork placement. Participants emphasize that traditional FW settings offered them more opportunities to develop “hard skills,” which increased self-efficacy when preparing to transition into the OT workforce. Participants echoed that they felt that virtual LEAP placements limited their learning and emphasized the perception that they missed out in learning the hard or clinical skills:

“*the hard skills of interventions and assessments, right? Because we’re not doing it, right, or it’s not– I did a– I did some assessments here and there for, like, the program evaluation if you can even call it that, but the– like, for example, a, a, a hard skill in a– in a rehab placement or in an acute care placement would be like using the mobility aids, right? The transfers, right, learning the transfers. . . it’s kind of drilled in your head over and over again, but actual practical use of [tranfers] and, hand hygiene strategies and you know, equipment use, all of the stuff that you are directly doing with the patient or client one-on-one, right? Charting, right? All of these skills that you need as a– as a health provider, as a practitioner. But in this placement, I– it just wasn’t there for me to actually gain and learn*”—1007

### Fieldwork environment

Participants shared that the setting of the LEAP placement (ie., in-person, virtual, and hybrid environments) had impact on their self-efficacy. It was discovered that the location of the placement environment (virtual, hybrid, in-person), and alternative service delivery methods significantly influenced their learning. Many participants did not prefer the virtual environment, with one noting the lack of opportunities for clinical skill development. Another participant shared that their online experiences hindered their ability to interact with clients. Yet another participant explained that limited in-person contact negatively impacted their self-efficacy, leaving them feeling unprepared for clinical practice.

*when the placement is virtual, it, it kind of-how to say? It kind of takes away the time from the clinical–the ability to do clinical practical skills.*—*1008*

*It’s hard because anything in health care you’re working with people, right? You’re, you’re face-to-face with a person who’s going through some sort of issue or challenge, and they need help, and you develop those skills by being beside them and listening to them and working with them. And if you don’t have that over the course of– or at least not as much, you’re gonna feel less prepared*.—*1002*

Despite the fact that this was a strong sentiment expressed by most participants, Some participants were able to reconcile with and appreciate the benefits of LEAP placements being online.

*. . .I think I’m just so trained for screens, and I’m totally comfortable on screens. So to put a positive on that [virtual LEAP placements], I’m pretty comfortable on screens.*—*1013*

#### Hybrid environment

Some participants had the opportunity to have a blended placement environment where half of their placement was online, and the other half was in person. One participant describes their experience learning in the hybrid learning environment:

*[. . .] my fourth placement is kind of a good example of how, a good compromise between the two ’cause my fourth placement was a hybrid. So I was supposed to be in-person for half the time and then online for the other half. But I found that when I actually had real in-person clinical experience and then my online portion was based off of what I was actually learning in person, I found. . .it a lot more relevant to what I was actually doing [. . .] I felt that. . ., the resources and the things I was working on actually contributed to my in-person clinical experience and contributed to the clinic in a tangible way, essentially. [. . .]I think the hybrid is, a good compromise. –1012*

#### Innovation

Innovation in a LEAP placement was interpreted as novel to participants. There are various markers of what innovative means to students. Participant interviews capture differences in innovation based on the program description of a LEAP placement and approaches and tools used to guide therapy. Moreover, aspects such as treatment approaches and resources used were deemed innovative to the participants. That includes the use of new tools and resources to support therapeutic interventions or the creativity required to adapt an occupation considering a client’s cultural context and norms were interpreted as innovative. For example, a participant narrative highlights adaptations to their approach for doing a shower assessment as part of an international LEAP placement, noting that:

*”A lot of times they may not have a shower with a spout, that goes downwards. They use a bucket and, are pouring water on themselves. So you have to modify how you even think about an occupation with the different individuals from different environments and countries” –1011.*

## Discussion and implications

This study sought to investigate and make meaning of the experiences of occupational therapy students and their perceived development of self-efficacy in non-traditional LEAP fieldwork placements. Findings indicate that self-efficacy development during LEAP placements was shaped by several factors. First, lack of role clarity detracted from self-efficacy development, while the opportunity for self-direction enhanced it. Second, the perception of preceptor support (e.g. level of support, preceptor role, and preceptor availability) could serve to both increase as well as decrease the development of self-efficacy. Third, the perceived value of the learning experience had mixed effects. Finally, the structure of the placement environment, whether virtual, hybrid, or in-person, also influenced and detracted from self-efficacy.

Our study found that student’s perceptions about their role and expectations of the student OT on fieldwork to be an influence on their self-efficacy. For example, participants who experienced lack of clarity regarding their role and responsibilities perceived this as negatively impacting their self-efficacy. Due to the nature of a LEAP placement, there may be no existing OT roles within the organization. Therefore, students are expected to create their own role and responsibilities specific to OT within the organization’s structure. It may be that students did not develop sufficient clinical or practical skills in their previous placements that were transferable to the unique nature of LEAP placements. This finding is in line with other studies suggesting that self-efficacy increases in later placements, providing students with a greater sense of security after undergoing more clinical experiences (Dancza et al., 2019, Derdall et al., 2002; Swinehart and Meyers, 1993). Another explanation may be due to students’ pre-existing baseline self-efficacy. Students who believe themselves to be more competent, in general, may be better suited to making decisions that help them excel within an academic and clinical setting and therefore excel at placement in spite of a lack of role clarity and their expectations of placement. These findings are congruent with the other literature (Bonsaksen, 2015; Kirke et al., 2007; Rodger et al., 2011) that explains the cyclical relationship between a positive learning environment and student self-efficacy.

Preceptor perception and support are key findings from this study as participants who believed they were well supported generally had higher perceptions of self-efficacy during fieldwork, congruent with other studies (Rodger et al., 2011). This aligns with Bandura’s (1982, 2012) view that external validation and feedback encourage personal improvement, thus contributing to positive growth in self-efficacy. Students who had off-site preceptors or preceptors who were not an OT did not have this same experience and perceived a negative impact on their self-efficacy. This may be due to the inability of the preceptor to provide relevant or timely feedback, which aligns with other literature (e.g. Kirke et al., 2007; Mulholland et al., 2006; Rodger et al., 2011) emphasizing feedback and its implication on effective student learning. Similarly, additional support, such as a fieldwork partner or other supervisors, increased self-efficacy. It may be that students felt a sense of comradery with their fieldwork partner and more comfortable around other supervisors due to factors such as age and level of experience in the field, despite not being an OT by background. This is supported by some studies (e.g. Kirke et al., 2007; Rodger et al., 2011) explaining the importance of preceptor rapport in self-efficacy development on fieldwork and also Bandura’s views of modeling, learning from others, and external validation as supporting positive growth in self-efficacy. However, more studies are required to further support this finding.

The transition to virtual environments during COVID-19 impacted students’ self-efficacy. Students who adapted well and valued the skills developed in their virtual LEAP placements had higher self-efficacy compared to those who preferred different environments or found the virtual setting suboptimal for learning. Viewing challenges, such as a new, unfamiliar or innovative environment, as opportunities aligns with Bandura’s (1982, 2012) perspective that reframing one’s outlook constructively can have positive impacts on self-efficacy development. To enhance self-efficacy, students might benefit from hybrid LEAP placement models, which combine virtual and in-person opportunities, allowing them to develop essential client-facing skills more effectively through different placement challenges.

Contrary to the findings of Derdall et al. (2002), our study found that students’ perceived self-efficacy is enhanced by practice settings aligned with their passions and interests. According to Bandura (1982, 2012), motivation and engagement drive the development of self-efficacy, which aligns with this finding. When LEAP placements and students’ interests were aligned, students were more engaged and motivated to be active learners, leading to growth in self-efficacy. In contrast, Derdall et al. (2002) found no relationship between practice setting, population served, choice in fieldwork placement, and OT students’ perceived self-efficacy, suggesting that self-efficacy is not tied to specific practice characteristics. This discrepancy may be due to practice contexts in the early 2000s, when virtual OT services and placements were not yet conceived as possibilities for service delivery. Additionally, Derdall et al. (2002) focused on traditional placements, while the current study examines LEAP placements, which require students to develop skills in non-traditional OT environments.

Different environments offer OT students unique learning opportunities in unique ways that contribute to their self-efficacy. LEAP placements have the potential to enrich the OT students’ learning experience by exposing them to unconventional settings, unveiling new opportunities to address gaps and enhance organizations that may not have previously recognized the benefits of OT services and thereby contribute to students’ professional and personal development. This is consistent with other research that examines OT students in community-based role-emerging practice placements. Golos and Tekuzener (2021) demonstrated that OT students placed in kindergartens, community mental health centers, and senior centers, have enhanced self-efficacy through exposure to diverse challenges and allowing them to develop and apply their skills in innovative contexts contributing to increased confidence and professional growth among students. Therefore, it suggests that LEAP placement environments may foster learning opportunities by placing students in contexts that demand creative application and utilization of treatment approaches, and resources that in turn, may support increased student self-efficacy.

There are limitations to this work. Given this was a secondary analysis, it was not possible to know the students’ pre-placement feelings of self-efficacy related to fieldwork competency development. The study took place during the initial year of the COVID-19 pandemic, a time of great uncertainty and fear for many, and this impact on participants was not captured in this analysis. Nonetheless, we believe this study provides a foundation for further exploration in relation to student perceptions of LEAP placements in relation to the development of self-efficacy.

### Conclusion

Our findings suggest that aspects of LEAP placements, during COVID-19, may have impacted student’s perceived self-efficacy in both positive and negative ways. Self-efficacy may have been positively impacted in LEAP placements that cultivated supportive environments and provided opportunities for practicing transferable skills while lack of role clarity appears to be associated with negative impacts on perceived self-efficacy. Students who demonstrated interest in a placement area were more motivated and described positive impacts on self-efficacy. Future research should seek to understand the relationship between specific types of supports and the fit between student interest and placement setting regarding self-efficacy development.

**Key findings**

- Non-traditional placements, that cultivate supportive environments, help to foster student self-efficacy.
- Providing opportunities for practicing transferrable skills is important to self-efficacy development in non-traditional placement settings.

**What the study has added**

This study demonstrates that occupational therapy students develop perceived self-efficacy in non-traditional fieldwork placements with available, supportive preceptors and environments that are aligned with their interests.
